# Supplementary material for: Distinct features of the regenerating heart uncovered through comparative single-cell profiling
Source: Biol Open. 2024 Apr 5;13(4):bio060156. doi: 10.1242/bio.060156 (PMC11007736; doi:10.1242/bio.060156)
Supplement: Supplementary information [file biolopen-13-060156-s1.pdf]

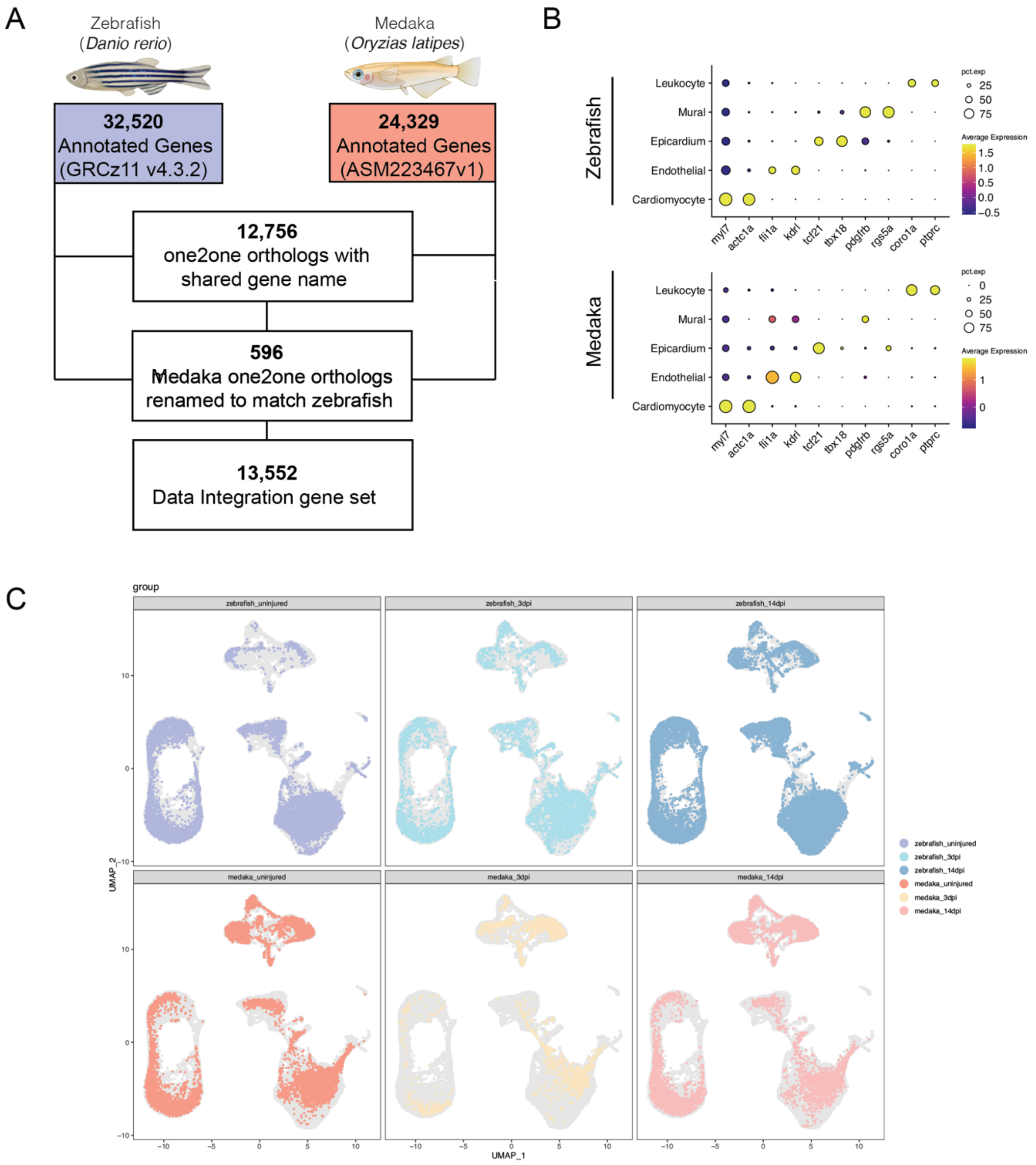

Fig. S1. Related to Fig. 1. A single-cell atlas of cardiac injury response in zebrafish and medaka. (A) Overview of data integration scheme. (B) Dot plot of cardiac cell type marker gene expression split by species. (C) UMAP embeddings showing distribution of each group of samples.

Biology Open • Supplementary information

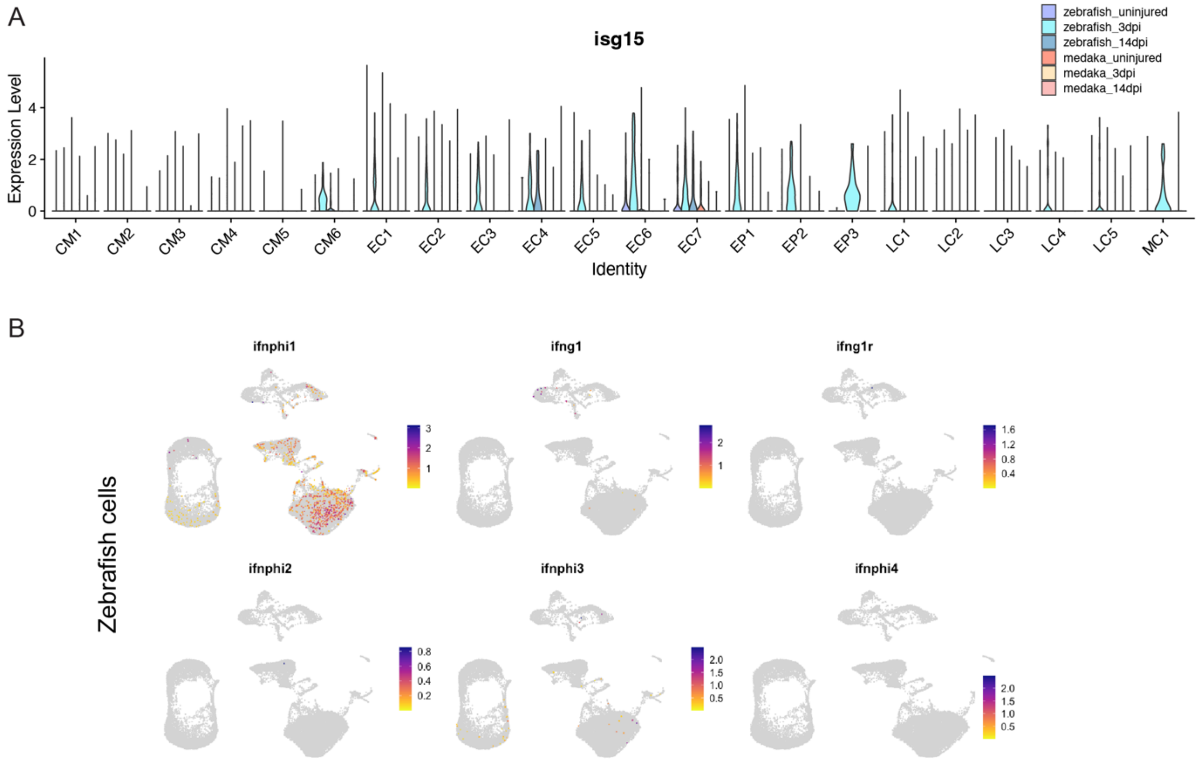

C

Medaka Gene	Putative Zebrafish Ortholog	ncells expressing
ENSORLG00000023723	ifnphi4	0
ENSORLG00000025524	ifnphi4	0
ENSORLG00000029917	ifnphi4	0
ENSORLG00000028380	ifnphi4	0
ENSORLG00000027228	ifng1	152

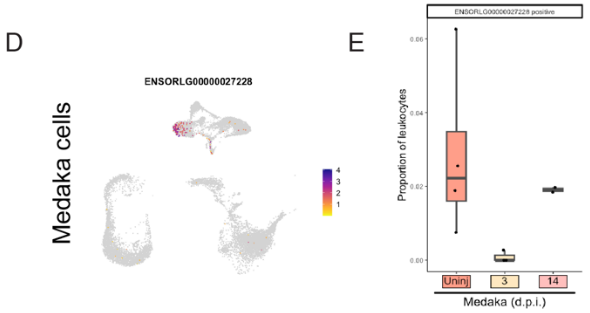

Fig. S2. Related to Fig. 2. Medaka lack an endogenous injury-induced interferon response. (A) Gene expression violin plot for *isg15* in each cell cluster, grouped by species and injury status. CM = cardiomyocyte, EC = endothelial cell, EP = epicardial cell, LC = leukocyte, MC = Mural Cell. (B) Gene expression feature plots of zebrafish cells for each annotated interferon isoform in the zebrafish genome color scale = expression level. (C) Medaka ensembl genes with predicted orthology to zebrafish interferon genes. (D) Gene expression feature plot of putative *ifng* ortholog

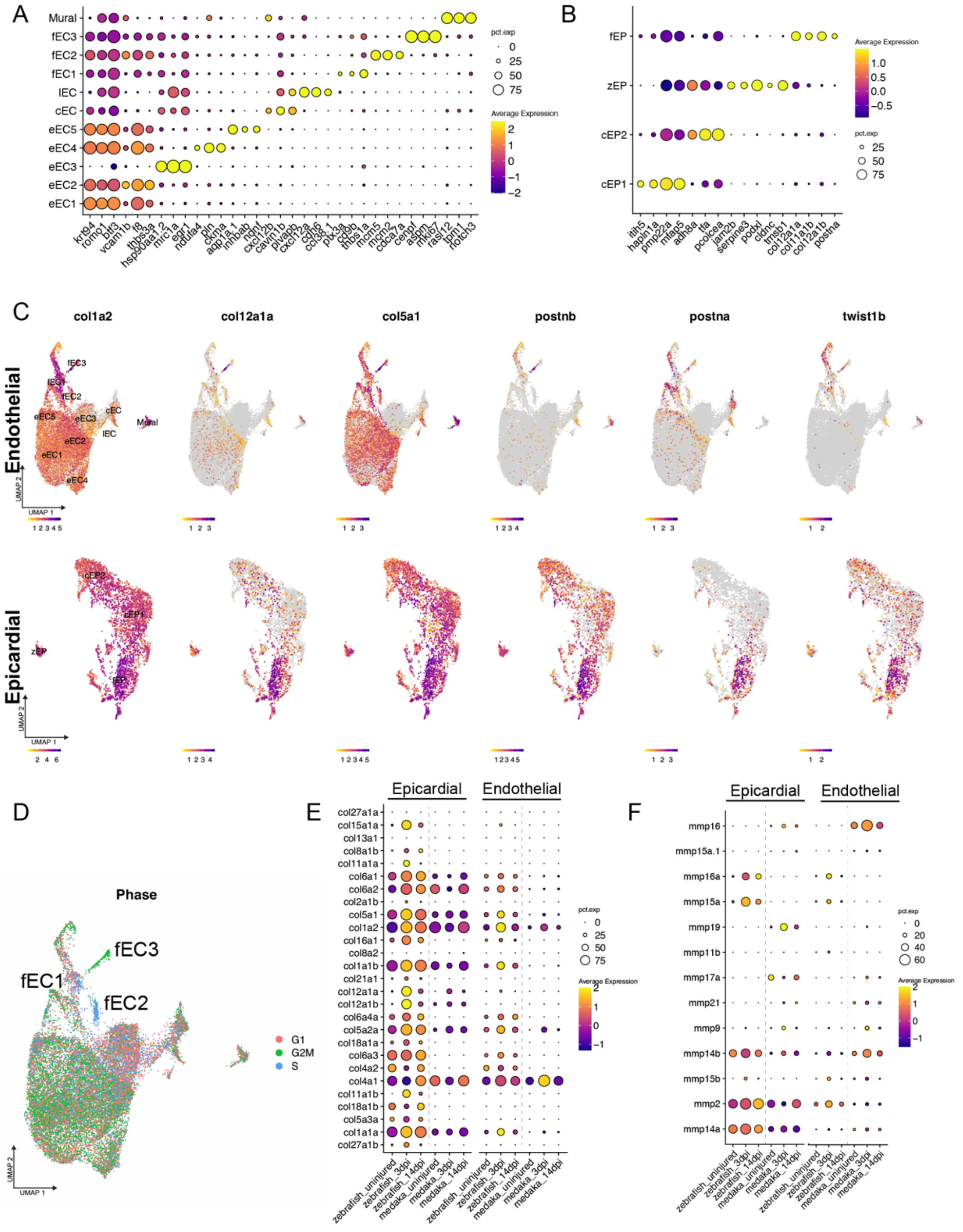

Fig. S3. Related to Fig. 4. Zebrafish and medaka share a partially overlapping fibrotic response to injury.

(A-B) Top markers for each individual (A) endothelial/mural or (B) epithelial cell cluster. (C) Gene expression feature plots for the indicated fibrosis genes in both epicardial (top) or endothelial (bottom) cell types.(D) UMAP embedding of endothelial cells classified by Seurat cell cycle scoring assignments highlighting uniform G2M/S phase assignments in fEC3 and fEC2, respectively. (E-F) Gene expression dot plots for all endothelial and epicardial cells for all (E) collagen and (F) matrix metalloproteinases expressed in >150 cells at the indicated time point and species.

Biology Open • Supplementary information

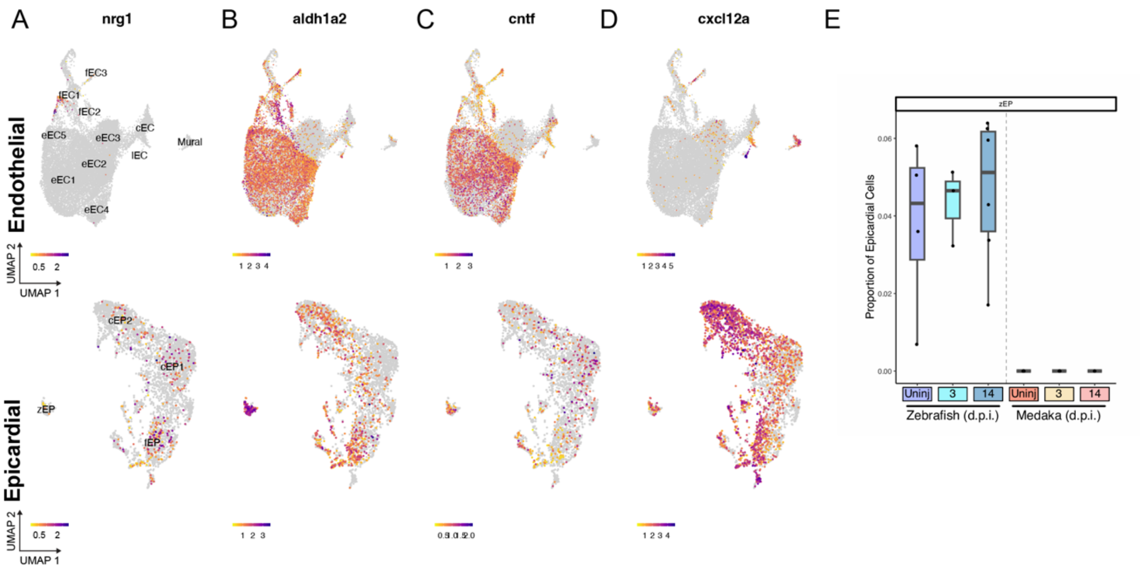

Fig. S5. Related to Fig. 4. Medaka epicardial and endothelial cells fail to express many pro-regenerative signals.(A-D) Gene expression feature plots of endothelial cells (top) and epicardial cells (bottom) showing normalized gene expression of (A) *nrg1,* (B) *aldh1a2,* (C) *cntf,* and (D) *cxcl12a* in integrated objects of both species. (E) Quantification of proportion of epicardial cells clustered as zEP at each time point in each species.

Table S1. Cardioprotective gene functions, related to Fig. 7

Gene	Effect	Citation
Valosin Containing Protein (*vcp*)	Promotes cardiomyocyte survival through preservation of mitochondrial integrity	Lizano *et al.* 2013
Chloride intracellular channel protein (*clic4*)	Maintains Ca^{2+} homeostasis after injury and promotes cell survival	Ponnalagu *et al.* 2022
Tropomodulin-1 (*tmod1*)2/12/24 10:12:00 PM	Involved in regulating cytoskeletal dynamics after injury, promoting cardiomyocyte migration.	Poch *et al.* 2022
Heat shock protein b1 (*hspb1*)	Protects myofibrils during cardiac stress	Brundel *et al.* 2008
Lactate dehydrogenase A (*ldha*)	Involved in metabolic reprogramming and promotes CM proliferation	Chen et al. 2022
Sarcolemma associated protein a (*slmapa*)	Provides mechanical support to cardiomyocyte membranes	Nader 2019
T-cadherin (*cdh13*)	Protects against cardiac injury via interaction with adiponectin and AMPK signaling	Denzel et al. 2010
succinate dehydrogenase complex, subunit A (*sdha*)	Upregulated in regenerating border zone regenerating cardiomyocytes, involved in metabolic reprogramming.	Spelat et al. 2022
Sarcoglycan delta (*sgcd*)	Stabilizes cardiomyocyte membrane in response to stress via ECM interaction.	Morikawa et al. 2015
